# Supplementary material for: Isolation and Functional Characterization of a LEAFY Gene in Mango (Mangifera indica L.)
Source: Int J Mol Sci. 2022 Apr 2;23(7):3974. doi: 10.3390/ijms23073974 (PMC9000169; doi:10.3390/ijms23073974)
Supplement: Supplementary file 1 [file ijms-23-03974-s001.zip › 7. ijms-1635223-supplementary (1).pdf]

TABLE S1 Primers used in this study

Primer ID	Primer sequence (5′-3′)	Application
LFY-F	ATGGATCCTGAAGCTTTCAC	Full length cloning
LFY-R	TTAGAAAGGCAGCTGATCAG	Full length cloning
MiLFY-F	TTCCCGGGATGGATCCTGAAGCTTTCACG	Vector construction
MiLFY-R	CCTCTAGATTAGAAAGGCAGCTGATCAGC	Vector construction
QLFYu	CGAAGGCGTCACATACTGTCT	qRT-PCR in mango
QLFYd	CACTTCACCAGGCTCAGTCA	qRT-PCR in mango
MiActin1u	CCGAGACATGAAGGAGAAGC	qRT-PCR in mango
MiActin1d	GTGGTCTCATGGATACCAGCA	qRT-PCR in mango
MiLFYu	AGGCTGGGGCAAGCTATATT	Positive plants detection
MiLFYd	ATACCAAATGGCCAGACGAG	Positive plants detection
AtActin 2 F	TCAGATGCCCAGAAGTCTTGTTCC	qRT-PCR in transgenic plants
AtActin 2 R	CCGTACAGATCCTTCCTGATATCC	qRT-PCR in transgenic plants
AtSOC1-F	CGAGCAAGAAAGACTCAAGTGTTTAAGG	qRT-PCR in transgenic plants
AtSOC1-R	TTCATGAGATCCCCACTTTTCAGAGAG	qRT-PCR in transgenic plants
AtAP1-F	CACCAAATCCAGCATCCTTAC	qRT-PCR in transgenic plants
AtAP1-R	GTTCGAGATCATTCCTCCTCA	qRT-PCR in transgenic plants
AtFTu	CTTGGCAGGCAAACAGTGTATGCAC	qRT-PCR in transgenic plants
AtFTd	GCCACTCTCCCTCTGACAATTGTAGA	qRT-PCR in transgenic plants
GUSu	CAACGAACTGAACTGGCAGA	qRT-PCR for *GUS* gene
GUSd	GCTAGTGCCTTGTCCAGTTG	qRT-PCR for *GUS* gene

Note: *AtActin* (No.AT3G18780), *AtSOC1* (No. AY007726), *AtAP1* (No. At1g69120), *AtFT* (No. AB027504)
